# Coordinated Regulation of Mesenchymal Stem Cell Migration by Various Chemotactic Stimuli

**DOI:** 10.3390/ijms21228561

**Published:** 2020-11-13

**Authors:** Donghyun Nam, Aran Park, Maria Jose Dubon, Jinyeong Yu, Wootak Kim, Youngsook Son, Ki-Sook Park

**Affiliations:** 1 Department of Biomedical Science and Technology, Graduate School, Kyung Hee University, Seoul 02447, Korea; ndh0721@naver.com (D.N.); kimwotak@khu.ac.kr (W.K.); 2 Graduate School of Biotechnology, Kyung Hee University, Yongin 17104, Korea; arvi2114@khu.ac.kr (A.P.); mariajosedubon@gmail.com (M.J.D.); jinyeong90@khu.ac.kr (J.Y.); ysson@khu.ac.kr (Y.S.); 3 East-West Medical Research Institute, Kyung Hee University, Seoul 02447, Korea

**Keywords:** mesenchymal stem cell, migration, substance P, TGF-β, SDF-1

## Abstract

Endogenous bone marrow-derived mesenchymal stem cells are mobilized to peripheral blood and injured tissues in response to changes in the expression of various growth factors and cytokines in the injured tissues, including substance P (SP), transforming growth factor-beta (TGF-β), and stromal cell-derived factor-1 (SDF-1). SP, TGF-β, and SDF-1 are all known to induce the migration of bone marrow-derived mesenchymal stem cells (BM-MSCs). However, it is not yet clear how these stimuli influence or interact with each other during BM-MSC mobilization. This study used mouse bone marrow-derived mesenchymal stem cell-like ST2 cells and human BM-MSCs to evaluate whether SP, TGF-β, and SDF-1 mutually regulate their respective effects on the mobilization of BM-MSCs. SP pretreatment of ST2 and BM-MSCs impaired their response to TGF-β while the introduction of SP receptor antagonist restored the mobilization of ST2 and BM-MSCs in response to TGF-β. TGF-β pretreatment did not affect the migration of ST2 and BM-MSCs in response to SP, but downregulated their migration in response to SDF-1. SP pretreatment modulated the activation of TGF-β noncanonical pathways in ST2 cells and BM-MSCs, but not canonical pathways. These results suggest that the migration of mesenchymal stem cells is regulated by complex functional interactions between SP, TGF-β, and SDF-1. Thus, understanding the complex functional interactions of these chemotactic stimuli would contribute to ensuring the development of safe and effective combination treatments for the mobilization of BM-MSCs.

## 1. Introduction

Endogenous mesenchymal stem cells are mobilized from the bone marrow to the peripheral blood and peripheral tissues in response to injury [1] or various pathological conditions including tumors [2,3], bone fractures [4], osteoporosis [5], and burns [6]. The injured and pathological tissues secrete various factors such as substance P (SP) [1], transforming growth factor-beta (TGF-β) [7,8,9,10], stromal cell-derived factor-1 (SDF-1) [11], and hepatocyte growth factor (HGF) [12], which induce the mobilization of bone marrow-derived mesenchymal stem cells (BM-MSCs).

SP is an 11-amino acid neuropeptide that mediates pain perception and the mobilization of BM-MSCs, which contribute to the regeneration of injured tissues [1]. SP activates several intrinsic signaling molecules, including extracellular signal-regulated kinases (ERKs) and protein kinase B (Akt) that mediate the mobilization of BM-MSCs [13]. TGF-β is synthesized in an inactive, latent form, which is converted to its active form in response to vascular injury, and active TGF-β induces the migration of BM-MSCs [9,10]. TGF-β ligands transduce signals via heterodimer receptors composed of type I and type II receptors [14], and then in response to TGF-β binding, the constitutively activated type II receptors initiate the activation of type I receptors [14]. These activated type I receptors then phosphorylate Smad2/3 proteins, which form a complex with Smad4 to regulate the transcription of TGF-β target genes [15]. Smad protein-dependent signaling is the canonical pathway for TGF-β signaling but TGF-β also transduces signals via ERKs, Akt, and p38, independently of Smad (noncanonical pathway) [15]. Both the canonical and noncanonical pathways are required for the migration of BM-MSCs [8]. SDF-1 (also known as CXCL12) binds to CXCR4, a G-protein coupled receptor that plays an important role in the mobilization of BM-MSCs [16]. CXCR4 mediates cellular migration via the activation of various signaling molecules including PI3K, ERKs, and Akt [17].

The regeneration of the injured tissues depends on the regenerative capacity of the migrated stem cells. It is very likely that injured tissues secrete a mixture of cytokines and growth factors, each of which has its own ability to mobilize BM-MSCs into the injury site. Understanding the combined effects of various factors on the mobilization of BM-MSCs is essential in determining the final level and mobilization kinetics of BM-MSCs to injured tissues. However, it is still unclear whether the various factors can modulate their respective mobilization capacities to fine-tune the mobilization of BM-MSCs. Additionally, SP, TGF-β, or SDF-1 can be applied to prime BM-MSCs for migration to enhance or maintain the therapeutic potential of these cells [18,19]. In this case, it is important to know whether pretreatment with SP, TGF-β, or SDF-1 affects the migration of the BM-MSCs in response to the other two factors. We used murine bone marrow-derived mesenchymal stem cell-like cells (ST2 cells) and human BM-MSCs to evaluate whether SP, TGF-β, and SDF-1 mutually regulate their respective BM-MSC migration-promoting abilities.

## 2. Results

### 2.1. Migration Capacity of ST2 and BM-MSCs in Response to TGF-β Is Impaired by SP Pretreatment

**Figure 1:**
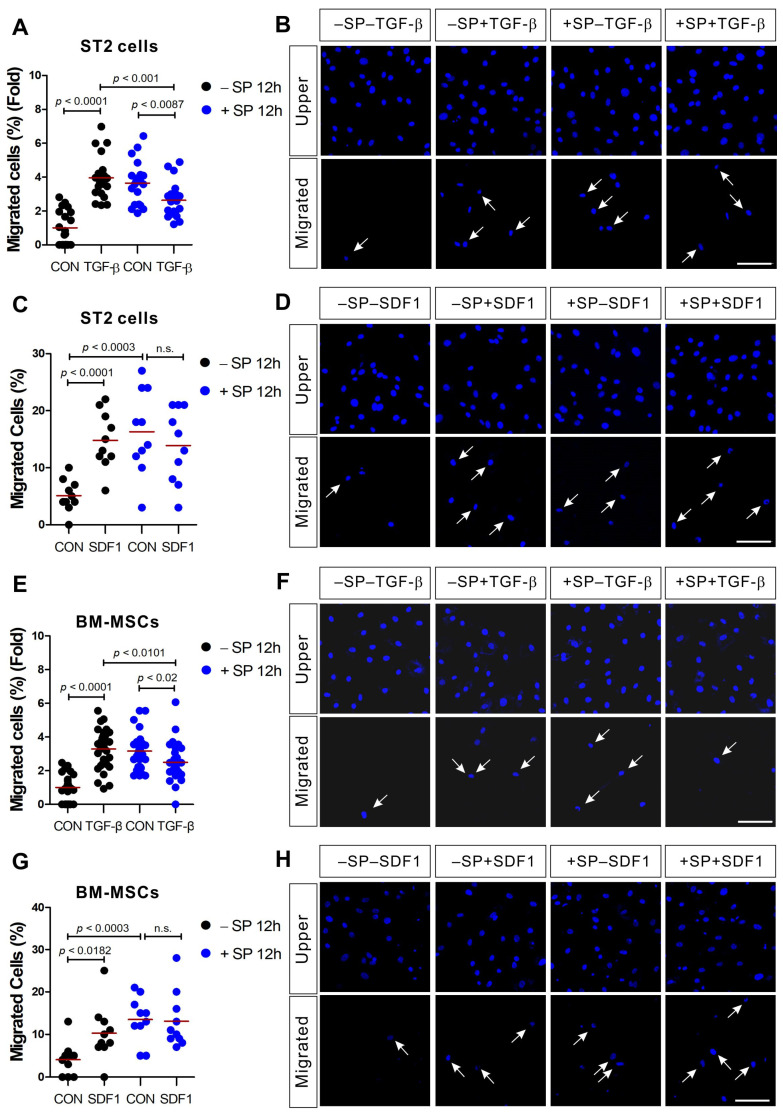
ST2 cells and bone marrow-derived mesenchymal stem cells (BM-MSCs) treated with substance P (SP) exhibited impaired migration in response to transforming growth factor-β (TGF-β), but not stromal cell-derived factor-1 (SDF-1). ST2 cells or BM-MSCs were pretreated for 12 h with SP (+SP; blue dots) or solvent (−SP; black dots) prior to their application in a transwell migration assay using TGF-β ((**A**,**B**) for ST2; and (**E**,**F**) for BM-MSCs) or SDF-1 ((**C**,**D**) for ST2; and (**G**,**H**) for BM-MSCs) as the stimulant. The control groups (CON) were treated with a solvent vehicle in all experiments. White arrows indicate the migrated cells on the lower membrane surface. Cells were stained with DAPI (blue) and the number of migrated cells is shown as a percentage of the total. The red lines indicate the mean value (*p* values were obtained by *t*-tests. n.s.; not significant) and the scale bar represents 100 µm.

As previously demonstrated [8,13], SP and TGF-β induce the chemotactic migration of ST2 cells and when compared to the control, SP and TGF-β stimulation increase the migration of ST2 cells by 4.6-fold and 2.7-fold, respectively (Figure S1A–D). SDF-1 also increased the migration of ST2 cells, 3.7-fold, compared to the control (Figure S1E,F). BM-MSCs also migrated toward TGF-β and SDF-1 (Figure. 1; black dot controls vs. black dot TGF-β or black dot SDF-1). To investigate the effect of SP pretreatment on the migration of ST2 cells and BM-MSCs in response to TGF-β or SDF-1, ST2 cells and BM-MSCs were treated with SP for 12 h before performing the migration assays. Even in the absence of chemotactic signal, the number of migrating ST2 cells and BM-MSCs in the SP pretreatment group was greater than the number of migrating cells in the controls (Figure 1; black dot controls vs. blue dot controls). Surprisingly, SP pretreatment decreased the migration capacity of ST2 cells and BM-MSCs in response to TGF-β (Figure 1A,B,E,F) but it did not inhibit or enhance the effects of SDF-1 (Figure 1C,D,G,H).

### 2.2. SDF-1 Pretreatment Enhances the Migration Capacity of ST2 in Response to SP and TGF-β, But Does Not Affect the Migration Capacity of BM-MSCs

**Figure 2:**
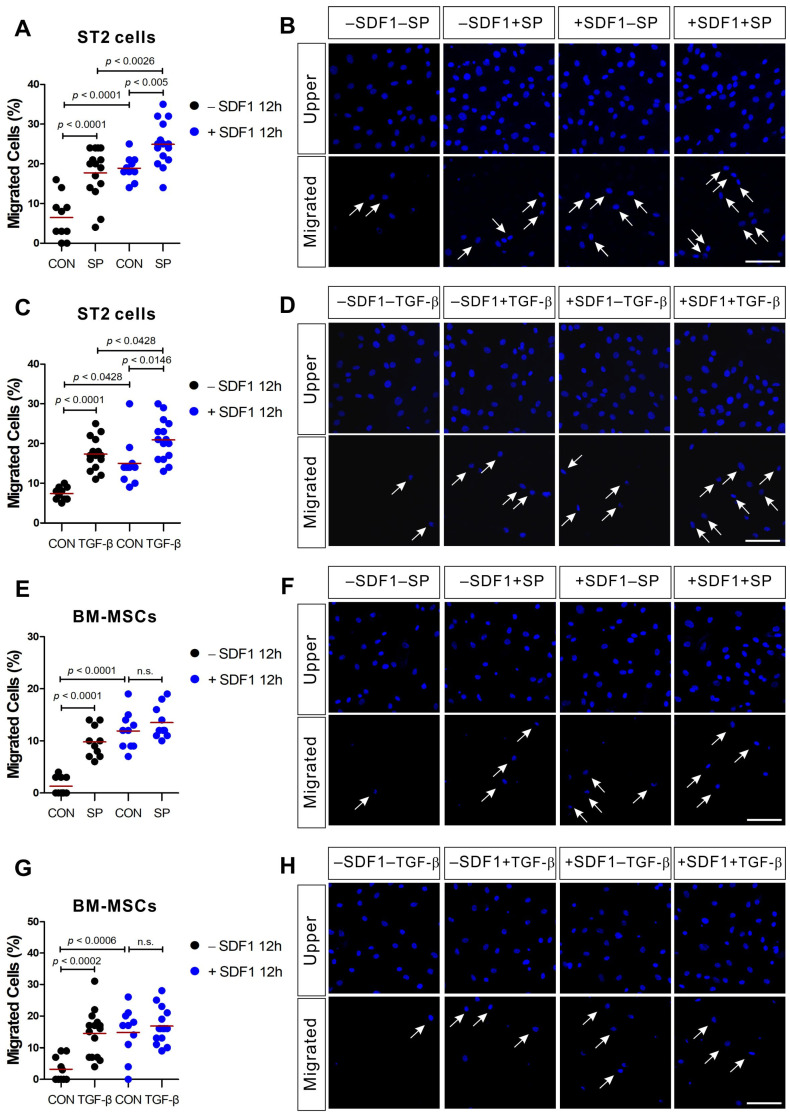
SDF-1 treatment enhances ST2 cell migration in response to SP and TGF-β, but not BM-MSC migration. ST2 cells or BM-MSCs were pretreated for 12 h with SDF-1 (+SDF1; blue dots) or solvent (−SDF1; black dots) prior to their application in a transwell migration assay using SP ((**A**,**B**) for ST2; (**E**,**F**) for BM-MSCs) or TGF-β ((**C**,**D**) for ST2; (**G**,**H**) for BM-MSCs) as stimulant. The control groups (CON) were treated with a solvent vehicle. White arrows indicate the migrated cells on the lower membrane surface. Cells were stained with DAPI (blue) and the number of migrated cells is shown as a percentage of the total. The red lines indicate the mean value (*p* values were obtained by *t*-tests. n.s.; not significant) and the scale bar represents 100 µm.

ST2 cells and BM-MSCs migrated toward SP and TGF-β (Figure 2; black dot controls vs. black dot SP or black dot TGF-β). We evaluated the effects of SDF-1 pretreatment on the migration capacity of ST2 cells and BM-MSCs in response to SP or TGF-β. SDF-1-pretreated ST2 and BM-MSCs showed increased motility even in the absence of other chemotactic signals when compared to the control cells (Figure 2; black dot controls vs. blue dot controls). In addition, a higher number of SDF-1-pretreated ST2 cells migrated toward both SP and TGF-β when compared with the control (Figure 2A–D; blue dot controls vs. blue dot SP or blue dot TGF-β). However, SDF-1-pretreated BM-MSCs did not exhibit any change in their response to SP (Figure 2E,F) or TGF-β (Figure 2G,H) chemotactic signals. It is unclear why BM-MSCs responded to the SDF-1 pretreatment differently from ST2 cells.

### 2.3. TGF-β Pretreatment Inhibits the Migration of ST2 and BM-MSCs in Response to SDF-1

**Figure 3:**
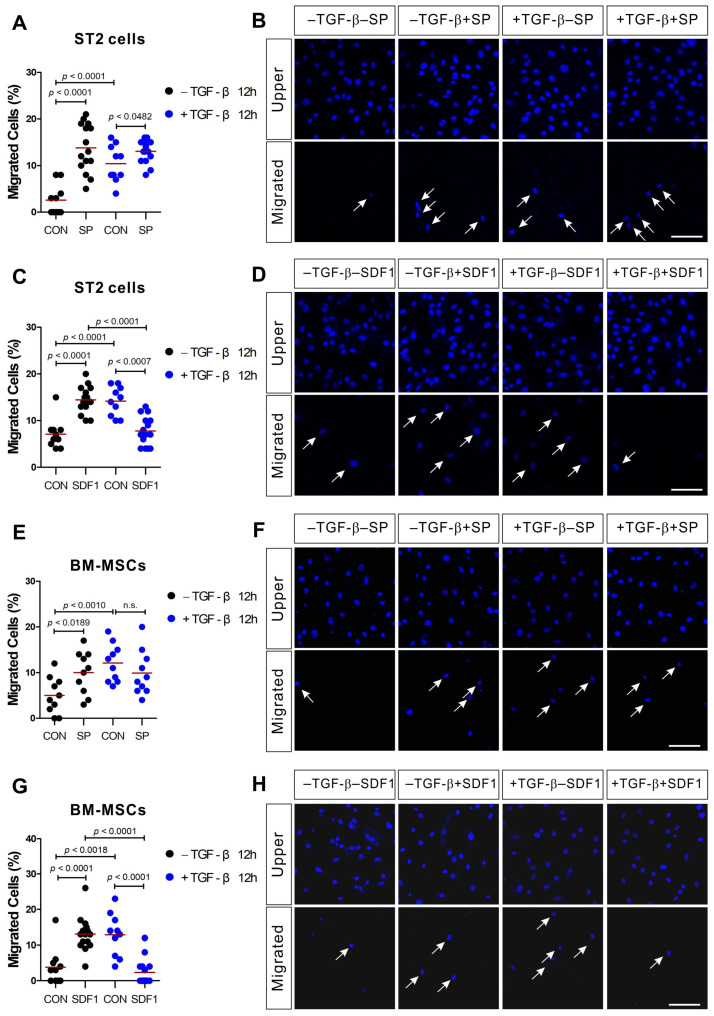
ST2 cells and BM-MSCs treated with TGF-β exhibit impaired migration in response to SDF-1, but not SP. ST2 cells or BM-MSCs were pretreated with TGF-β (+TGF-β; blue dots) or solvent (−TGF-β; black dots) for 12 h prior to their application in a transwell migration assay using SP ((**A**,**B**) for ST2; (**E**,**F**) for BM-MSCs) or SDF-1 ((**C**,**D**) for ST2; (**G**,**H**) for BM-MSCs) as the stimulant. The control groups (CON) were treated with a solvent vehicle. White arrows indicate the migrated cells on the lower membrane surface. Cells were stained with DAPI (blue) and the number of migrated cells is shown as a percentage of the total. The red lines indicate the mean value (*p* values were obtained by *t*-tests. n.s.; not significant) and the scale bar represents 100 µm.

We went on to evaluate the effect of TGF-β pretreatment on the cellular migration of ST2 and BM-MSCs in response to SP or SDF-1. TGF-β pretreatment increased the migration potential both of ST2 and BM-MSCs, which is in agreement with our SP and SDF-1 data. TGF-β pretreatment increased the number of the migrating cells even when there was no chemotactic gradient (Figure 3; black dot controls vs. blue dot controls), but TGF-β pretreatment did not significantly affect the response of ST2 and BM-MSCs to SP (Figure 3A,B,E,F; blue dot controls vs. blue dot SP). However, TGF-β pretreatment did reduce the response of ST2 and BM-MSCs to SDF-1 stimulation (Figure 3C,D,G,H; blue dot controls vs. blue dot SDF-1), although TGF-β did not decrease CXCR4 (SDF-1 receptor) mRNA expression (unpublished).

### 2.4. The SP Receptor Antagonist Rescues SP Pretreatment-Mediated Inhibition of ST2 and BM-MSC Migration in Response to TGF-β

**Figure 4:**
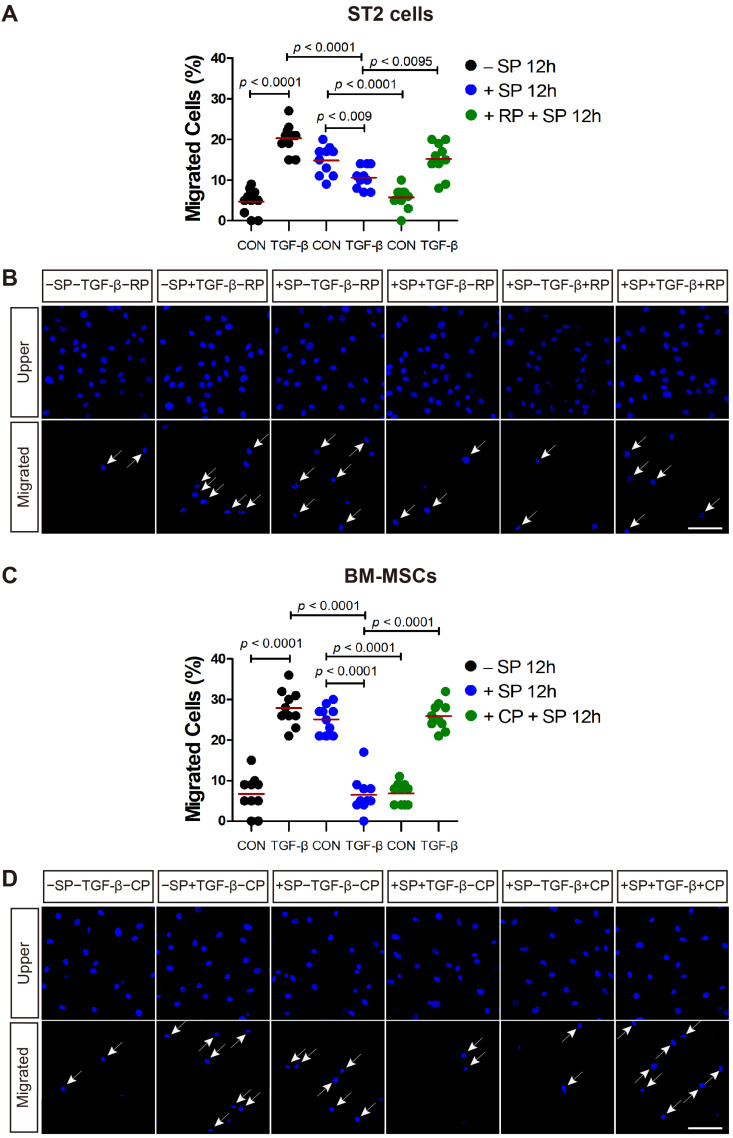
The SP receptor antagonist rescues the cell migration in response to TGF-β. (**A**,**B**) RP 67580 (RP; SP receptor antagonist; green dots) rescued the migration capacity of SP-pretreated ST2 cells in response to TGF-β stimulation. (**C**,**D**) CP-96345 (CP; SP receptor antagonist; green dots) rescued the migration capacity of SP-pretreated BM-MSCs in response to TGF-β stimulation. White arrows indicate the migrated cells on the lower membrane surface. Cells were stained with DAPI (blue) and the number of migrated cells is shown as a percentage of the total. The red lines indicate the mean value (*p* values were obtained by *t*-tests) and the scale bar represents 100 µm.

The SP receptor, neurokinin-1 receptor (NK1R) is expressed on ST2 cells and BM-MSCs. SP-mediated signaling can be effectively inhibited in murine cells and human cells by the NK1R antagonist RP 67580 and CP-96345, respectively [20]. SP pretreatment increased the motility of ST2 cells (Figure 4A,B; black dot control vs. blue dot control) and BM-MSCs (Figure 4C,D; black dot control vs. blue dot control) in the absence of chemotactic factors, but downregulated the migration of ST2 cells and BM-MSCs (Figure 4; blue dot control vs. blue dot TGF-β) in response to TGF-β, as evidenced by Figure 1. When RP 67580 was added to the ST2 cells prior to SP pretreatment, it inhibited the SP pretreatment-mediated increase in ST2 cell motility (Figure 4A,B; blue dot control vs. green dot control). Furthermore, RP 67580 partially rescued ST2 migration in response to TGF-β, which was downregulated in the SP pretreatment group (Figure 4A,B; blue dot TGF-β vs. green dot TGF-β). The SP pretreatment-mediated increase in BM-MSCs cell motility was also inhibited by NK1R antagonist CP-96345 (Figure 4C,D; blue dot control vs. green dot control). Moreover, the migration of BM-MSCs in response to TGF-β, which was downregulated in the SP pretreatment group was rescued with CP-96345 (Figure 4C,D; blue dot TGF-β vs. green dot TGF-β).

### 2.5. SP Pretreatment Impairs Noncanonical TGF-β Signaling in ST2 and BM-MSCs

**Figure 5:**
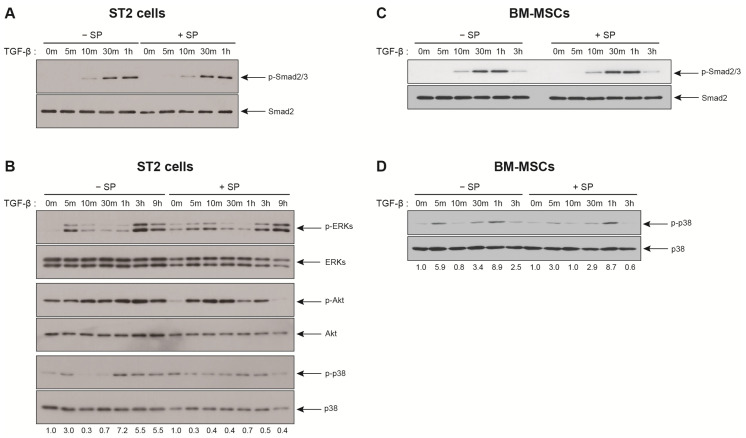
The SP pretreatment decreases p38 activation in response to TGF-β. (**A**,**B**) Western blot analysis of ST2 cells that been treated 10 ng/mL TGF-β for the indicated time intervals after pretreatment with SP (+SP) or solvent (−SP). (**C**,**D**) Western blot analysis of BM-MSCs that have been treated 1 ng/mL TGF-β for the indicated time intervals after pretreatment with SP (+SP) or solvent (−SP). Protein levels of total Smad2/3, ERKs, Akt, or p38 served as the internal control for phosphorylated Smad2/3 (p-Smad2/3), phosphorylated ERKs (p-ERKs), phosphorylated Akt (p-Akt), and phosphorylated p38 (p-p38), respectively. The band intensity of p-p38 was normalized against that of p38, and the ratios are shown (two independent experiments).

Then, we went on to speculate that SP pretreatment may prevent TGF-β-mediated signal transduction. Firstly, we checked whether SP pretreatment affected the TGF-β receptor-mediated canonical signaling in response to TGF-β. Regardless of SP pretreatment, Smad2/3 proteins were phosphorylated and the phosphorylation level of Smad2/3 in response to TGF-β was not affected by SP pretreatment (Figure 5A,C). Thus, it could be inferred that the expression levels of TGF-β type I/II receptors remain unchanged by SP pretreatment and the canonical TGF-β signaling pathway was not impaired in ST2 cells and BM-MSCs. Indeed, TGF-β type I receptor mRNA expression was not changed in BM-MSC in response to SP treatment (unpublished). Following this, the activation of ERKs, Akt, and p38 was examined since the activation of TGF-β noncanonical pathways, such as ERKs, Akt, and p38, is necessary for the migration of ST2 cells in response to TGF-β [8]. ERKs, Akt, and p38 were activated in ST2 cells in response to TGF-β treatment (Figure 5B). TGF-β-mediated activation of ERKs was not affected by SP pretreatment (Figure 5B). However, the kinetics of TGF-β mediated activation of both Akt and p38 changed upon the SP pretreatment. The extent of Akt activation in response to TGF-β following the SP pretreatment was observed to be higher when compared to a single treatment with TGF-β (Figure 5B; difference between 0 min and 10 min in the control group vs. difference between 0 min and 10 min in SP pretreatment group). However, TGF-β-mediated activation of p38 was inhibited by the SP pretreatment. Biphasic phosphorylation of p38 was observed at 5 min and late time points with TGF-β treatment and SP pretreatment decreased the biphasic activation of p38 in ST2 cells (Figure 5B). The SP pretreatment-mediated decrease in p38 activation might impair ST2 cells migration in response to TGF-β. Then, we examined whether the SP pretreatment affects TGF-β-mediated activation of p38 in BM-MSCs. TGF-β also stimulated the biphasic activation of p38 in BM-MSCs (Figure 5D). Interestingly, SP pretreatment downregulated TGF-β-mediated early phosphorylation of p38 in BM-MSCs, but not the delayed phosphorylation of p38 (Figure 5D).

## 3. Discussion

SP, which is secreted from injured tissues, mobilizes mesenchymal stem cells from the bone marrow to the injury sites, thereby contributing to the regenerative repair of injured tissues [1]. Injured tissues secrete additional factors including TGF-β [9,10] and SDF-1 [21,22,23], each of which induces the migration of both ST2 and BM-MSCs. However, whether mobilizers of BM-MSCs like SP, TGF-β, and SDF-1 can influence each other’s ability to induce the mobilization of mesenchymal stem cells has not yet been investigated. This study demonstrates that SP pretreatment impairs the migration of ST2 and BM-MSCs in response to TGF-β and that TGF-β pretreatment downregulates the migration of ST2 and BM-MSCs in response to SDF-1.

SP activates ERKs and Akt in ST2 cells, and this activation is required for ST2 cell migration in response to SP [13]. SDF-1 activates the ERKs, but it also activates Janus kinase 2/signal transducer and transcription 3 factor, inducing the migration of BM-MSCs [24]. TGF-β activates several signal transduction pathways in BM-MSCs via interactions with its Type I and II receptor complexes [8,14]. TGF-β noncanonical activation uses ERKs, Akt and p38 to transduce its signals while the canonical pathway uses Smad2/3-dependent signaling. Both the canonical and noncanonical pathways are responsible for TGF-β-mediated migration of ST2 and BM-MSCs [8]. Thus, we examined if SP pretreatment impaired TGF-β-mediated activation of the canonical and noncanonical pathways in ST2 cells and BM-MSCs since SP pretreatment impaired the migration of ST2 cells and BM-MSCs in response to TGF-β. The activation of the TGF-β canonical pathway, as shown by Smad2/3 phosphorylation, was not inhibited by SP pretreatment. In contrast, the treatment of ST2 cells and BM-MSCs with SP prior to TGF-β treatment downregulated p38 activation. Hence, the decrease in TGF-β-induced migration of ST2 cells and BM-MSCs may be a result of the impaired activation of p38 upon SP pretreatment. Therefore, further experiments are required to determine whether the SP pretreatment impairs the migration of ST2 cells and BM-MSCs in response to TGF-β via the impaired activation of p38. We also sought to determine whether TGF-β pretreatment impaired SDF-1 receptor CXCR4 in BM-MSCs since the TGF-β pretreatment impaired the migration of BM-MSCs in response to SDF-1. The TGF-β pretreatment did not decrease mRNA expression of SDF-1 receptor CXCR4 in BM-MSCs (unpublished). However, the TGF-β pretreatment may be able to regulate total protein level of CXCR4 and/or intracellular trafficking and endocytosis of CXCR4 independently of CXCR4 mRNA expression. Further experiments are required to investigate whether TGF-β regulates the surface expression of CXCR4 protein and SDF-1 signaling in ST2 cells and BM-MSCs.

Various factors produced by injured tissues mobilize mesenchymal stem cells from the bone marrow to the peripheral blood and later to the injured tissues, with each compound exhibiting their own dynamics and efficiencies. Many studies suggest that a combination therapy of different mobilization agents allows the safe and rapid mobilization of stem cells, especially hematopoietic stem cells and progenitors from the bone marrow [25,26,27]. Combination therapy of various BM-MSC mobilization agents is also being evaluated in an effort to enhance the mobilization of BM-MSCs in regenerative medicine. The ex vivo priming approaches use various biological, biophysical and pharmacological factors to improve the therapeutic potential of BM-MSCs in several applications including in anti-inflammatory and wound healing settings [19,28,29,30]. However, our study suggests that combination treatments using various BM-MSCs mobilizers may have antagonistic effects on BM-MSC mobilization. The priming of BM-MSCs using a specific mobilizer may downregulate BM-MSC mobilization in response to another mobilizing agent, thus more studies are required to understand the complex interactions of these signaling molecules to ensure the development of safe and effective combination treatments for the mobilization of BM-MSCs.

## 4. Materials and Methods

### 4.1. Cell Culture

ST2 cells were purchased from Riken Cell Bank (Tsukaba, Japan) and grown in Roswell Park Memorial Institute medium (RPMI 1640, Gibco-BRL, Grand Island, NY, USA) supplemented with 10% heat-inactivated fetal bovine serum (FBS, Gibco-BRL), 100 U/mL penicillin, and 100 μg/mL streptomycin (P/S, Gibco-BRL). Cells between passage 5 and 8 were used for all the experiments. BM-MSCs were obtained from Lonza (Basel, Switzerland) and maintained in Mesenchymal Stem Cell Growth medium (MSCGM; Lonza). Cells between passages 4 and 6 were used for all experiments. Dulbecco’s Modified Eagle’s medium (DMEM, GE Healthcare Life Sciences, Logan, UT, USA) supplemented with 10% FBS, 2 mM *L*-glutamine, and 100 U/mL P/S was used for all of the BM-MSCs migration experiments. All cells were maintained at 37 °C in a humidified incubator containing 5% CO_2_.

### 4.2. Transwell Migration Assay

ST2 cells (2.5 × 10^4^) or BM-MSCs (2 × 10^4^) were seeded on Millicell inserts (8 μm pore size; EMD Millipore, Billerica, MA, USA) coated with type I collagen (5 μg/mL; Nitta Gelatin NA Inc., Morrisville, NC, USA). After 6 h of incubation, the media was changed to DMEM (GE Healthcare Life Sciences) supplemented with 2% FBS and 100 U/mL penicillin. After overnight incubation, SP (300 nM), TGF-β (100 ng/mL for ST2 cells or 10 ng/mL for BM-MSCs), or SDF-1 (50 ng/mL) were added to the lower chamber of each well. After 12 h, the inserts were fixed using 4% paraformaldehyde in phosphate buffered saline (PBS) for 10 min at room temperature, washed, and then stained using 4′,6-diamidino-2-phenylindole (DAPI, Invitrogen, Carlsbad, CA, USA) for 10 min. Then the membrane inserts were mounted on slides using ProLong Gold antifade mounting solution (Invitrogen). Cells on either the lower or upper surface of the five randomly selected areas of each Millicell membrane were imaged using a Zeiss LSM 700 confocal microscope (Zeiss LSM700, Zeiss, Oberkochen, Germany) and experiments were done on duplicated samples for each experimental condition. The number of cells in each image was counted using Adobe Photoshop CS6 (Adobe Systems Incorporated, San Jose, CA, USA) and the number of migrated cells was determined as a percentage of the total cells on both sides of the insert. To evaluate the effects of pretreatment with SP, TGF-1, or SDF-1 on the migration of ST2 and BM-MSCs in response to either of the other two factors, the cells were serum starved for 18 h and treated with SP, TGF-β or SDF-1 for 12 h and then seeded as before for the migration assay. SP receptor antagonist, RP 67580 (10 nM) or CP-96345 (1 μM) was added to the ST2 cells or BM-MSCs prior to their SP pretreatment, respectively and then used in the migration assay as before.

### 4.3. Western Blot Analysis

ST2 cells or BM-MSCs pretreated with SP (300 nM) were seeded on 12-well plates at a density of 1.6 × 10^5^ cells per well using serum starved media. After 5–6 h of incubation, the cells were treated with TGF-β for various time intervals. Total protein lysates were prepared by adding 2 × SDS buffer (120 mM Tris-HCl (pH 6.8), 4% SDS, 0.02% bromophenol blue, 20% glycerol, 10% β-mercaptoethanol) directly into each of the wells. Following primary antibodies (Cell Signaling Technology, MA, USA) were used for western blot analysis: phospho-Smad2 (Ser465/467)/Smad3 (Ser423/425), phospho-p44/42 MAPK (ERKs) (Thr202/Tyr204), phospho-Akt (Ser473), phospho-p38 MAPK (Thr180/Tyr182), Smad2, p44/42 MAPK (ERKs), Akt, and p38 MAPK. Band densities were measured using ImageJ software v 1.53e (NIH, Bethesda, MD, USA).

### 4.4. Statistics

Quantitative data are presented as the mean ± standard deviation (SD). Comparisons between experimental groups were done using unpaired Student’s *t*-tests on GraphPad v. 5.01 software (GraphPad Software, San Diego, CA, USA; http://www.graphpad.com). Differences were considered statistically significant at *p* < 0.05.

## Supplementary Materials

The following are available online at https://www.mdpi.com/1422-0067/21/22/8561/s1, Figure S1: ST2 cells migrate in response to SP, TGF-β, and SDF-1.

## Abbreviations

BM-MSCs	Bone marrow-derived mesenchymal stem cells
NK1R	Neurokinin-1 receptor
SDF-1	Stromal cell-derived factor-1
SP	Substance P
TGF-β	Transforming growth factor-beta
