# Supplementary material for: Coordinated Regulation of Mesenchymal Stem Cell Migration by Various Chemotactic Stimuli
Source: Int J Mol Sci. 2020 Nov 13;21(22):8561. doi: 10.3390/ijms21228561 (PMC7696304; doi:10.3390/ijms21228561)
Supplement: Supplementary file 1 [file ijms-21-08561-s001.pdf]

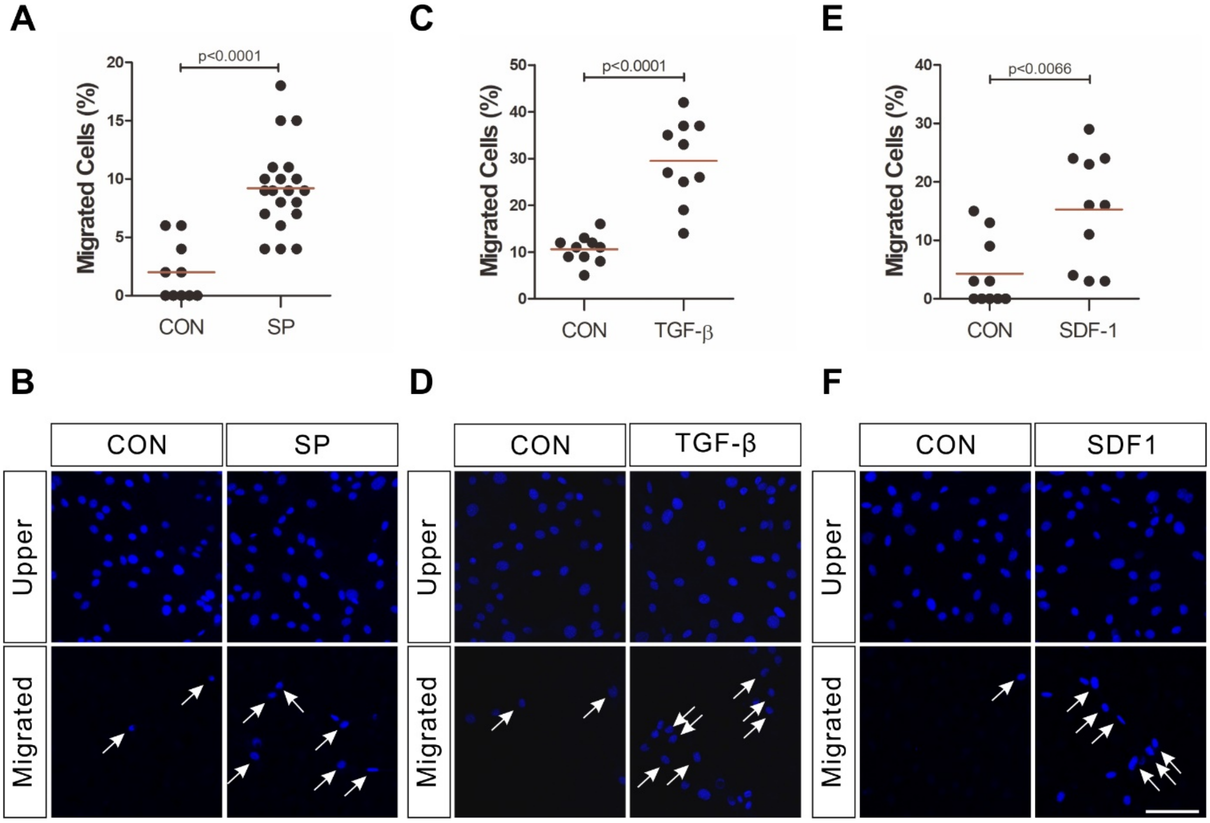

Supplementary Figure. S1: ST2 cells migrate in response to SP, TGF-β, and SDF-1. ST2 cells migrate after 12 h of treatment with (**A,B**) SP (300 nM), (**C,D**) TGF-β (100 ng/mL), (**E,F**) SDF-1 (50 ng/mL). The control groups (CON) were treated with a solvent vehicle control. ST2 cells were stained with DAPI (blue) and five fields of view from the lower and upper chambers of each Millicell well were imaged and counted. White arrows indicate the migrated cells present on the lower membrane surface. The red lines indicate the mean value (p values were obtained by t-tests) and the scale bar represents 100 μm.
